# Feeling our place in the world: an active inference account of self-esteem

**DOI:** 10.1093/nc/niae007

**Published:** 2024-04-01

**Authors:** Mahault Albarracin, Gabriel Bouchard-Joly, Zahra Sheikhbahaee, Mark Miller, Riddhi J Pitliya, Pierre Poirier

**Affiliations:** Département d'Informatique, Université du Québec à Montréal, 405 Rue Sainte-Catherine Est, Montreal H2L 2C4, QC, Canada; Département d'Informatique, Université du Québec à Montréal, 405 Rue Sainte-Catherine Est, Montreal H2L 2C4, QC, Canada; CHU Sainte-Justine Research Center, University of Montreal, 5757, Av. Decelles bureau 500, Montreal, QC H3S 2C3, Canada; Monash Centre for Consciousness and Contemplative Studies, Monash University, Melboume, Australia; Psychology Department, University of Toronto, Toronto, ON M5S 3G3, Canada; Department of Experimental Psychology Oxford University, Oxford OX1 2JD, United Kingdom; Département d'Informatique, Université du Québec à Montréal, 405 Rue Sainte-Catherine Est, Montreal H2L 2C4, QC, Canada

**Keywords:** self-esteem, active inference, model, sociometer

## Abstract

Self-esteem, the evaluation of one’s own worth or value, is a critical aspect of psychological well-being and mental health. In this paper, we propose an active inference account of self-esteem, casting it as a sociometer or an inferential capacity to interpret one’s standing within a social group. This approach allows us to explore the interaction between an individual’s self-perception and the expectations of their social environment.When there is a mismatch between these perceptions and expectations, the individual needs to adjust their actions or update their self-perception to better align with their current experiences. We also consider this hypothesis in relation with recent research on affective inference, suggesting that self-esteem enables the individual to track and respond to this discrepancy through affective states such as anxiety or positive affect. By acting as an inferential sociometer, self-esteem allows individuals to navigate and adapt to their social environment, ultimately impacting their psychological well-being and mental health.

## Introduction

The word “esteem” came into the English language in the 15th century, and it was associated with the evaluation ( i.e. to find the value) of some object or someone (Bailey 2nd, 2003). Then, the word “self” was prefixed to “esteem,’, wherein “self-esteem” referred to the evaluation of the self. William James (James, 1890) suitably introduced the term to the psychological literature, referring to the concept as the confidence and beliefs one has about their own abilities and worth (Abdel-Khalek, 2016).

The importance of self-esteem in our lives has been revealed by the exponential research conducted in various fields of psychology, including social psychology (Abrams and Hogg, 1988), developmental psychology (Wigfield et al., 1991b), cognitive psychology (Fennell, 1997), and psychiatry. In the realm of social psychology, self-esteem plays a crucial role in shaping our interactions and relationships with others. Healthy self-esteem can lead to healthier social connections, better conflict resolution, and overall greater satisfaction in our social lives (Farineau et al., 2011; Abe, 2004). On the other hand, low self-esteem can result in social anxiety, less assertiveness, and a higher likelihood of settling for unsatisfying relationships. In developmental psychology, self-esteem has been found to be a key factor in the healthy development of children and adolescents. It contributes to academic achievement, reduces the risk of problematic behaviors, and promotes resilience and overall well-being (Wigfield et al., 1991a; Connelly, 1993). Moreover, the development of self-esteem in early life stages can have long-lasting effects on individuals’ mental health and success in adulthood (Coffey and Warren, 2020). Self-esteem has also been identified as a key factor in the development and maintenance of many psychological disorders such as anxiety (Nima et al., 2013), depression (Hilbert et al., 2019), and narcissism (Di Pierro et al., 2016). Self-esteem thereby predicts well-being and being functional in life domains such as relationships, mental and physical health, and work (Orth and Robins, 2014).

Despite the evidence that self-esteem has important real-world consequences and is of considerable personal and societal significance, the mechanisms underlying self-esteem remain generally associated with other phenomena rather than cognitive mereology of the first principle components of self-esteem. Additionally, the mechanisms proposed underlying self-esteem do not seem to be integrated even though they have very compatible approaches.

The self-organizing self-esteem model (De Ruiter et al., 2017b) is one such key theory that provides a dynamic systems approach to understanding self-esteem. This model proposes that self-esteem is a self-organizing system that adapts and evolves in response to interactions with the environment. It emphasizes the importance of feedback loops and the interplay between an individual’s self-esteem and their social environment. The model suggests that self-esteem is not a static trait, but a dynamic process that is continually shaped and reshaped by our experiences and interactions.

However, a limitation of the self-organizing self-esteem model is that it may lack a formal mathematical framework to precisely capture the complex dynamics of self-esteem. It may not also fully integrate various aspects of self-esteem, such as the hierarchical structure of moment-to-moment variations and more stable trait self-esteem.

In line with this perspective, the aim of this paper is to address these research gaps by formalizing self-esteem using the active inference framework (AIF). Active inference provides a mathematical foundation that allows for a more precise modeling of the dynamic and self-organizing nature of self-esteem. By integrating several of the proposed models, including the self-organizing self-esteem model, we propose a nested temporal structure where, in the lower level, an agent experiences moment-to-moment variation of self-esteem states based on sensory observation, and at a higher hierarchical level, more stable trait self-esteem is modeled. This approach not only captures the dynamic nature of self-esteem but also provides a comprehensive and integrative understanding of how self-esteem develops and functions, addressing the limitations of previous models.

Self-esteem mechanism models are theories that explain how self-esteem is formed, maintained, and changed over time. Some of the most well-known self-esteem mechanism models are as follows:

- Social Identity Theory: This theory suggests that our self-esteem is largely based on the groups we belong to and the extent to which we identify with them (Tajfel and Turner, 1979).
- Self-Discrepancy Theory: This theory proposes that our self-esteem is influenced by the gaps between our actual self, ideal self, and ought self (Higgins, 1987).
- Sociometer Theory: This theory proposes that self-esteem functions as an internal monitor of our social acceptance and that it reflects our perceived degree of social inclusion or exclusion (Leary and Baumeister, 2000).
- The Self-Organizing Self-Esteem Model explains how an individual’s self-esteem self-organizes through the complex dynamic circular causality between trait and state self-esteem. Its authors reject traditional models where trait self-esteem causes state self-esteem as a contextual response to external stimuli. Instead, they offer a model where trait self-esteem, viewed as a landscape of attractor states, constrains, in a top-down fashion, the development of states and discrete experiences of self-esteem and is slowly built, in a bottom-up fashion, by the contextual occurrence of states and experiences of self-esteem (De Ruiter et al., 2017a).
- Self-Evaluation Maintenance Model: This theory proposes that our self-esteem is influenced by the closeness of our relationships with others and the extent to which we perceive them to be more or less successful than us in areas that are important to us (Tesser, 1988).
- Self-Determination Theory: This theory proposes that self-esteem is closely linked to our sense of autonomy, competence, and relatedness (Deci and Ryan, 2000).
- Cognitive Dissonance Theory: This theory suggests that our self-esteem is affected by the extent to which our thoughts and behaviors align with our self-concept (Festinger, 1957).
- Self-Perception Theory: This theory proposes that our self-esteem is shaped by our observations of our own behavior and the reactions of others to that behavior (Bem, 1972).
- Contingencies of the Self-Worth Model: This model suggests that our self-esteem is based on how we perceive our performance in certain domains that are important to us, such as relationships, academic achievement, or physical appearance (Crocker et al., 2003).
- Dual-Process Model: This model proposes that there are two different processes that contribute to our self-esteem: an automatic, implicit process that is based on our past experiences and a reflective, explicit process that is more conscious and deliberative (Epstude and Roese, 2008).

One noteworthy attempt to model self-esteem dates back to an article by Dubois and colleagues (DuBois et al., 1996), in which the research team assessed the impact of multifactorial variables, such as social and institutional contexts, on self-esteem. However, the paper does not attempt to provide a formal account of these interactions and therefore fails to enable precise assessments of self-esteem in an ecological niche.

Importantly, there are intricate relations between the individual’s self evaluation, the feedback the individual gets from the world following his or her actions, and ideals that are massively offered to the individual by the cultural niche and which act as norms on which to assess self-value.

We argue that self-esteem is related to an individual’s ability to anticipate and react appropriately to its sociocultural environment (see Sections “AIF/formalizing the self” and “Socially-Predicted Self” for a detailed explanation and defense and Section “Self-Esteem as Predicted Adaptivity” for an explicit active inference model). Various aspects of our proposal are supported by the models of self-esteem discussed earlier. For example, Self-Determination Theory proposes that self-esteem is closely linked to an individual’s sense of competence and its ability to act autonomously in their environment. Similarly, Cognitive Dissonance Theory suggests that discrepancies between an individual’s self-concept and its behavior can lead to lower self-esteem, as can perceptions of failure or incompetence in important domains (which aligns with the Contingencies of Self-Worth Model). Moreover, Self-Perception Theory proposes that individuals infer their self-esteem from observing their own behavior and the reactions of others to that behavior, which can influence an individual’s anticipation and reaction to its environment. The Dual-Process Model, for its part, suggests that self-esteem can be influenced by both implicit and explicit processes, which could include an individual’s automatic, intuitive responses to environmental cues as well as more reflective and deliberate reactions. Finally, like the more mechanistic details of our Active Inference Model, the Self-Organizing Self-Esteem Model proposes that trait self-esteem, which corresponds to our Active Inference Model’s set of learned self-esteem priors, both constrains state self-esteem, which corresponds to our model’s self-esteem predictions, and is built in a complex dynamical fashion out of each discrete self-esteem experience, which corresponds our model’s error predictions. By taking into account the dynamical, social, cognitive, emotional, and behavioral aspects of the models of self-esteem discussed earlier, we can conclude that an individual’s ability to anticipate and react appropriately to their environment can play a key role in the development and maintenance of self-esteem.

In Section “Introduction”, we will explain how the self is conceptualized in the active inference paradigm. In Section “AIF/formalizing the self”, we discuss the role of the social niche in modulating self-esteem. In Section “Socially-Predicted Self”, we introduce new research on the role of affectivity in active inference as a measure of the system adaptivity. We go on to show how self-esteem can be considered a reflection of changes in our social adaptivity within a certain niche. In the final section, we develop a new computational account of self-esteem.

## AIF/formalizing the self

Free energy principle, proposed by Friston, is a computational approach that explains how living systems maintain themselves within phenotypic or characteristic states (Friston and Stephan, 2007; Friston et al., 2009). It offers a framework that combines learning, perception, action, and decision-making. This principle involves two key objective functions: the variational free energy, which describes the fitness of the biological agent’s internal world model of the environment (mapping latent states of the agent to sensor observations and inferring the dynamics of the environment), and the expected free energy, which selects a potential future set of actions by balancing the maximization of extrinsic values (defined in terms of prior preferences) and the epistemic value (exploration to reduce uncertainty). The Free Energy Principle and the Active Inference can be contextualized within a Partially Observed Markov Decision Process (POMDP) framework, which accommodates both uncertainty and decision-making with incomplete information. The agent employs a mean-field approximation to form beliefs about hidden states based on proprioceptive and exteroceptive information. Within active inference, policy selection in AIF is considered as Bayesian model selection, where the agent chooses a set of actions (policy *π*) which are predicted to minimize the expected free energy in the future. The variational free energy of internal states and the expected free energy can be defined respectively as follows:


(1) \begin{equation*}\begin{split} F[Q(s_{1:T},\pi)]&= \\ &\;\;\underbrace{{D}_{\mathrm{KL}}\Big[Q(s_{1:T},\pi)||P(s_{1:T},\pi)\Big]}_{\text{complexity}}\\ &-\underbrace{\mathbb{E}_{Q(s_{1:T},\pi)}\Big[\ln P(o_{1:T}|s_{1:T},\pi)\Big] }_{\text{accuracy}} \end{split}\end{equation*}

(2) \begin{equation*}\begin{split} P(\pi)=\sigma(-G(\pi))&=\sum_{\tau}G(\pi,\tau)\\ G[\pi,\tau]&= \\ &\quad\mathbb{E}_{\tilde{Q}}\left[\ln Q(s_{\tau}|\pi)-\ln P(o_{\tau},s_{\tau}|o_{1:T},\pi)\right]\\ &=\underbrace{\mathbb{E}_{\tilde{Q}}\left[\ln Q(s_{\tau}|\pi)-\ln Q(o_{\tau}|s_{\tau},\pi)\right]}_{\text{epistemic value}}\\ &-\underbrace{\mathbb{E}_{\tilde{Q}}\left[\ln P(o_{\tau})\right]}_{\text{extrinsic value}}\\ &=(\mathbf{A}\mathbf{s}_{\pi}^{\tau})\cdot\left(\ln \mathbf{o}_{\pi}^{\tau}-\ln\mathbf{C}^{\tau}\right)+\mathbf{s}_{\pi}^{\tau}\cdot\mathbf{H}, \end{split}\end{equation*}

where *P* is the probability distribution that represents a generative model responsible for generating a series of observations ($o_{1:T}$). On the other hand, *Q* encodes to the agent’s beliefs, which approximates the posterior distributions of the latent causes ($s_{1:T}$) that underlie the sensory data in a given environment. Equation (1) captures two crucial components: the first term aims to minimize assumptions beyond the prior and seeks a compelling explanation for the data. In essence, it utilizes the Kullback-Leibler divergence (${D}_{\mathrm{KL}}$) to measure the deviation between posterior beliefs regarding states given policies and their corresponding prior beliefs. It emphasizes minimizing the divergence between prior beliefs and new observations, ensuring that updates to these beliefs should be parsimonious, thereby maintaining a close alignment with the original priors. The second term assesses the goodness of fit between the generative model and the observed data. One can compute posterior expectations by estimating the derivative of the variational free energy (Eq. 1) with respect to the sufficient statistics of the latent causes and setting it to zero


(3) \begin{equation*}\begin{split} \partial_{\mathbf{s}}F_{\pi}&=\frac{\partial F[Q(s_{1:T},\pi)]}{\partial\mathbf{s}_{\pi}^{\tau}}=0\\ \bar{\mathbf{s}}_{\pi}^{\tau}&=\sigma (\underbrace{\ln \mathbf{B}_{\pi}^{\tau-1}\mathbf{s}_{\pi}^{\tau-1}}_{\text{past}}+\underbrace{\ln \mathbf{B}_{\pi}^{\tau}\cdot\mathbf{s}_{\pi}^{\tau+1}}_{\text{future}}+\underbrace{\ln \mathbf{A}\cdot\mathbf{o}^{\tau}}_{\text{present}}), \end{split}\end{equation*}

where *σ* is a Softmax function, **A** is a likelihood mapping, and $\mathbf{B}_{\pi}$ is a transitions between hidden states over time. This leads to Bayesian belief updates, which are informed by counterfactual beliefs about future states and postdictive beliefs about past states, as well as by the observed consequences of different possible plans or policies (Friston et al., 2017). This framework can be interpreted as a model of perception, wherein the process of inferring hidden states (represented by posterior expectations $\mathbf{s}_{\pi}^{\tau}$) provides the most plausible explanation for observed outcomes, based on the past (prior expectations) and possible future states.

Equation (2) provides insight regarding beliefs about policies that can only rely on future outcomes. Here, we have $\tilde{Q} = Q(s_{\tau}|\pi)P(o_{\tau}|s_{\tau})$. In this equation, the cybernetic system minimizes the expected free energy, which allows the agent to deduce a set of actions facilitating Bayes optimal behaviors. It is important to note that this approach highlights the significance of exploration as an essential component in the process of selecting an optimal policy. This framework is used to demonstrate how self-esteem emerges with these mechanics (Friston, 2023). In the second equality, $\mathbf{A}\;\mathbf{s}_{\pi}^{\tau}$ expresses policy-specific outcomes and $\mathbf{C}^{\tau}$ represents a vector of preferences over outcomes. The first term quantifies risk as the expected cost of a policy in terms of a divergence from the preferred outcome, while the second term corresponds to ambiguity, where **H** denotes the relative entropy or uncertain mapping to observations.

**Figure 1.**
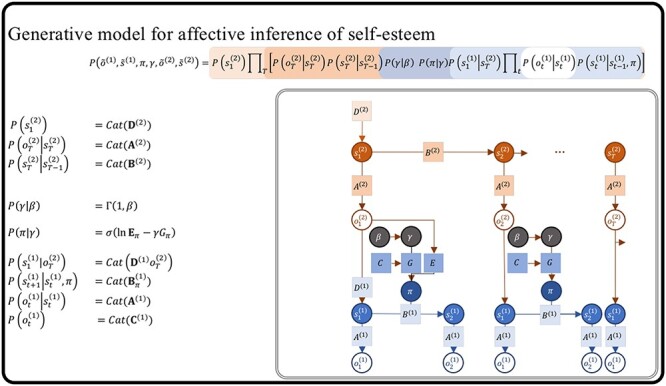
Hierarchical message-passing architectures of self-esteem. The top equation illustrates this process, employing a mean field approximation to approximate posterior beliefs. This approximation involves the product of marginal distributions over hierarchical levels and points in time. The generative model represents the joint probability of observed outcomes and their latent causes. The **D**^*i*^ arrays demonstrate the initial state $s_{1}^{i}$, given the state of the level above, and they are referred to as the empirical priors. The probability of an outcome given hidden states is called the likelihood (**A**^*i*^). Next, we define matrix **B**^*i*^ as the transition probabilities of the next state given the current state contingent on policies *π*. The preference vectors **C**^*i*^ encode the degree of desirability of associated outcomes or preferences. The policy is sampled from a Gibbs distribution or softmax function of the path integral of expected free energy (*G*) multiplied by with inverse temperature or precision *γ*. The temperature parameter modulates the difference between the expected free energy under posterior and prior beliefs regarding policies.

In Figure 1, an individual, referred to as an “agent,” organizes information about themselves and their environment into a hierarchical “generative model.” The generative model consists of internal world models that capture expectations about future events and the behavior of objects and individuals (known as intuitive physics and intuitive psychology). The agents take actions based on their latent states in order to achieve their desired preferences (Török et al., 2022). By utilizing a POMDP model, individuals can anticipate future states and make informed decisions to reach their objectives. This model enables predictions about future observations by capturing complexity and details across different levels. At the lower levels of the generative model, the individual perceives sensory data about the world and maps them to possible causal states. The agent can generate data that provide strong evidence for the internal model, which is referred to as some form of self-evidencing (Hohwy, 2007). This structure can be extended to nested hierarchies where inferred states can become observations for further inference as we move up in the generative model (Hesp et al., 2021). As information ascends through the hierarchy, latent states capture more abstract concepts, such as the notion of self. As information travels down, the higher levels serve to set priors over the lower levels. Consequently, this hierarchical structure manifests as both temporally deep levels of inference and coalesces into patterns at higher and more complex levels of inferences, correlated with slower timescales Kirchhoff et al. (2018); Limanowski and Blankenburg (2013); Veissiére et al. (2020).

The generative model plays a crucial role in shaping agents’ beliefs about the hidden states of the world, while policies implicitly encode expectations regarding future outcomes. In modelizations, the “**D** vectors” contain the prior probabilities over initial latent states, $P(s_1)$. These probabilities are continually adjusted during the learning process through evidence accumulation by counting the number of times an initial state occurs (Fig. 1). By calculating the expected “free energy” for the future, which is influenced by the value of a target distribution (represented by the “**C** matrix”), individuals can gather evidence for realistic strategies that align with their goals and phenotypic preferences. For instance, the **C** matrix could encapsulate a preference for positive reinforcement and feedback from colleagues at work, supportive interactions within close relationships, or acceptance and recognition within one’s social circle.

In the inferential process proposed by AIF, an individual can understand the states of the world by either actively updating their internal world model to match observations or by acting to alter their environment (Friston et al., 2016). In goal-directed planning, an agent evaluates the potential value of future rewards given the current situation and employs prediction to foresee the consequences of potential plans. Conversely, habitual behaviors are rooted in the information, which is slowly accrued over time as a collection of cached action values or utilities (Maisto et al., 2019). While goal-directed planning requires more cognitive effort, it offers greater flexibility compared to the rigidity of habitual behavior.

Decision-making processes in AIF reconcile these two behaviors by sampling from a softmax function, a Gibbs distribution, which balances habitual (**E**) and goal-directed (***G***) influences. The posterior expectation over policies after an observation is computed using the softmax function, described by the following expression:


(4) $$ Q(\pi) = \sigma\big(\ln \mathbf{E} - \boldsymbol{F}_{\pi,\tau}- \gamma\;\boldsymbol{G}_{\pi,\tau}\big). $$

In this equation, the **E** vector comprises past expectations about the selection of strategies, or policies, given specific contexts. These expectations, which serve as habitual priors, are formed through the computation of expected free energy (***G***) for each policy, allowing for an effective management of cognitive resources in stable environments. The temperature parameter, ***γ***, influences the confidence level in the expected free energy calculations, thereby adjusting the stochastic nature of the decision-making process. Specifically, ***γ*** encapsulates the agent’s subjective estimation of how well its internal model and resultant policies are aligned with the external world. Notably, in the context of the initial distribution over policies prior to making any observations (*π*_0_), $\boldsymbol{F}_{\pi,\tau}$ is set to zero.

Free energy is a concept borrowed from physics, specifically the second law of thermodynamics, which deals with the natural tendency toward disorder. When applied to biological systems, free energy provides insights into the understanding of how these systems manage incoming information from environments. Free energy bounds the long-term average over surprises of systems that maintain their homeostasis (Friston et al., 2009). For example, a system will encounter surprise when it does not adequately predict a consequence to a sequence of events. When it inaccurately predicts this sequence, it may not be prepared to deal with it appropriately. For example, a bunny unexpectedly encountering a bear will not survive if it simply continues on its path as if nothing had changed. This surprising information needs to be integrated into the model and factored in for future choices.

The free energy principle constrains any theory for action and perception in goal-directed cybernetic systems. The system selects a series of actions or strategies to modify sensory data by using a method that minimizes free energy, known as Bayesian model selection (Parr and Friston, 2017). By mentally simulating counterfactual scenarios, one can identify preferred outcomes based on the consequences of actions and adopt behaviors that reduce uncertainty and expected free energy in the future. Crucial for minimizing free energy in the present moment is the capability of the system to determine how free energy is likely to evolve over time in various context and relative to the system’s behavioural possibilities.

In the active inference literature, the body is considered to be the primary prior and a crucial factor in understanding the model of self, others, and the world (Allen and Tsakiris, 2018). Expanding on this perspective, Safron (2021) emphasizes that the self is not a separate entity but rather emerges from embodied interactions and experiences within the social environment. Based on these ideas, an embodied self-model was introduced, which incorporates a hierarchical predictive memory system and a cybernetic controller for individuals. Taking this concept further, we can view self-esteem as relatively stable states at a higher level of hierarchy, functioning as a set of empirical priors. These priors are learnable posterior expectations that guide an individual’s value-driven actions within the system, moment by moment. In essence, self-esteem serves as a foundation that influences how individuals perceive themselves and navigate social interactions. For example, in any social setting, the state of self-esteem or self-concept can modulate the precision of the **B** matrix at a lower level. Individuals with higher self-esteem tend to have more precise expectations about the outcomes of their behaviors, whereas those with lower self-esteem may have less precise expectations and exhibit more volatile behavior in a given context.

Agents refine their self-esteem traits, which function as empirical priors, when exposed to novel experiences over time. Synaptic weights—akin to Dirichlet concentration parameters—accumulate evidence, enhancing their predictive accuracy according to Hebbian plasticity principles. Neurons co-activating in response to specific states and observations strengthen their connections, facilitating the agent’s predictive capabilities and behavioral responses within their environment. This neuronal activity encodes the divergence between predictions from empirical priors and observed sensations (prediction errors), cast as gradient flow, driving message passing and, consequently, belief updating in superficial pyramidal cells at higher cortical levels. Moreover, variations in the precision of synaptic gains influence belief updating and determine the attentional selection of observations, which in turn affects the excitability or postsynaptic gain of neuronal populations broadcasting prediction errors (Friston, 2023). This modulation can either attenuate or strengthen the updates to prior beliefs, depending on whether the precision increases or decreases, respectively (Table 1).

**Table 1.** Visual representation of the Bayesian brain model through active inference.

	Higher-ordered beliefs	
Conditional posterior expectations $\mathrm{Cat}(\mathbf{B}|\mathbf{b})$	Hierarchical neuronal message passing	Prior beliefs $\mathrm{Dir}(\mathbf{b})$
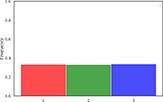	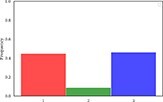	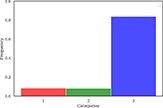
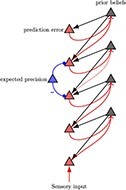	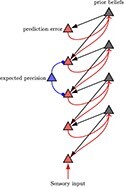	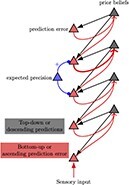
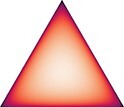	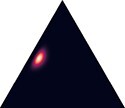	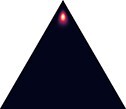

The left column displays the conditional posterior expectations ($\mathrm{Cat}(\mathbf{B}_{\pi}|\mathbf{b})$), illustrating how empirical priors are updated into posterior beliefs through exposure to new data. The middle column shows the process of Hierarchical Neuronal Message Passing, depicting the flow of prediction errors and their resolution through expected precision adjustments. The right column represents prior beliefs ($\mathrm{Dir}(\mathbf{b})$), with Dirichlet distributions reflecting the strength and certainty of empirical priors before sensory input. The rows illustrate different levels of precision of prior beliefs (from low at the top to high at the bottom), which develop through learning. This model demonstrates the cognitive process by which agents update their beliefs and adjust their behavior, emphasizing the interplay between prediction, precision, and learning in the brain’s hierarchical structure.

### Self as self-prediction

Recent advances in computational neuroscience have begun to be successfully applied to the study of the “self” (Metzinger, 2004; Seth et al., 2012; Allen and Friston, 2018; Apps and Tsakiris, 2014; Ishida et al., 2015). The self, as understood in the context of active inference, is a probabilistic concept that is based on an agent’s ability to process self-related information through a multimodal hierarchical cortical network designed to approximate Bayesian inference (Seth and Tsakiris, 2018a). In this paper, the authors propose that the self can be understood as a process of prediction error minimization, in which the brain generates predictions about its own states and the states of the environment and then minimizes the error between these predictions and the actual sensory input it receives. The self is therefore viewed as a probabilistic construct that is constantly being updated and refined through the process of prediction error minimization. This process is used to preserve the agent’s integrity against the entropic pressures of the environment. The self is constantly updated as the agent perceives itself in the world through the accumulation of available sensory evidence.

Thus, selfhood can be formalized as an emergent property of the brain’s hierarchical predictive processing. The self is not a static entity but a dynamic, probabilistic construct continuously updated through the minimization of prediction error. This process is mathematically encapsulated by the variational free energy *F* and expected free energy *G* equations, which govern the agent’s interactions with the world. The self is not only a probabilistic construct updated through sensory evidence but also intimately connected with the body’s visceral rhythms, shaping affective perception and self-consciousness (Allen et al., 2022).

Building on these principles, our model extends the understanding of self within this hierarchical framework. We emphasize the role of self-concept predictions at varying levels of complexity and their continuous refinement through sensory interaction. These predictions are then compared with the actual sensory information we receive from our environment. Any differences between our predictions and the actual sensory information are considered errors, which help us refine our self-concept (Seth and Tsakiris, 2018b). The resulting error signals are used to update the agent’s prior beliefs and generative models. The self-model plays a key role when agents perceive themselves in the world as “an experiencing subject” by constantly updating this relation through accumulating available sensory evidence.

### Cognition and recursive observation

The system does not simply model the outside world, but it also models itself to infer its own internal states. As we have established, as information travels up and down, it carries meta-properties. Thereby, the agent must model how well it calibrates itself in order to act how it intends in the external world and bring about the external states it prefers (Limanowski, 2017).

In this sense, we can talk about meta-cognition. We already have examples of this phenomenon (Badcock et al., 2019). Specifically, attentional control has been shown to require meta-cognition through modeling where attention is, and where attention should be, all while also modeling the world itself (Sandved Smith et al., 2020). Cognition is not a constant and infallible process, and the agent must also observe itself inferring, in order to evaluate the accuracy of its observations and calibrate its own processes. The nested structure of cognition allows for awareness and concentration. Indeed, cognition functions in layers of signals and optimization of these signals. The system receives input and must infer the cause of this input. As the layers communicate up and down, the agent is able to focus more efficiently. The agent can then give an updated weight to its inferences, which will be reevaluated based on the outcome of the predictions.

By observing its own process, the system is able to give a precision value to its own predictions and adapt its policies according to the most likely outcome to limit free energy spending. A prediction with a low precision will potentially be less likely to be trusted. Such predictions would have less of an effect on any potential update of the model or change in behavior for the agent to fit the predictions. Most cognitive processes function in this nested fashion, partly due to the structure of the cortex, which is itself a nested hierarchical structure (Feinberg, 2011). Input from the lower levels gets weighted and clustered. These clusters are themselves clustered and weighted as they go up the cognitive chains (Hipólito et al., 2021). The peculiarity of the Bayesian process is that this input is not the origin of cognition, so much as the evidence against which the predictions are either confirmed or updated.

In Sandved-Smith et al. (2021), metacognition is monitoring and control of one’s own cognitive processes. In a hierarchical model, higher levels provide priors for the lower levels, effectively creating a feedback loop for self-regulation and adjustment of cognitive processes based on their precision or confidence. This is mathematically instantiated by precision parameters that weight the prediction errors at various levels of the hierarchy, modulating the influence of sensory evidence versus prior beliefs.

Precision weighting can also be understood as the degree to which an individual’s self-perception is influenced by the mismatch between their perceived and actual social standing. For instance, the precision of prediction errors in the AIF can be related to the individual’s confidence in their self-perception and their perceived social feedback. The more precise the prediction errors, the more the individual will trust their self-perception over the mismatch with social expectations.

It is pertinent to explore the connection of self-esteem and meta-cognition with broader frameworks of personality and neurobiology, especially in the context of hierarchical, goal-oriented systems (Safron and Sheikhbahaee, 2023). Metacognition, involving the reflection and regulation of one’s thoughts and behaviors, intersects closely with personality traits of stability and plasticity. These traits, as suggested by the Cybernetic Big 5 Theory, are key to understanding self-regulatory meta-cognitive strategies in cybernetic systems, which are sculpted as minimal description lengths and manifest as stable emergent equilibria, shaping ecological adaptations as traits (Waddington, 1942). The interplay of underlying personality traits, as influenced by neuromodulation, and meta-cognitive processes, is prominent in this context. In this regard, the dynamic tension between stability and plasticity in personality as key meta-traits in personality resonates with the variability in self-esteem as individuals navigate and find their place within the social hierarchy. For example, high self-esteem correlates with stability and higher precision in interoceptive and proprioceptive signals, leading to stable and deterministic action selection and a tendency toward extrinsic/pragmatic values. Conversely, low self-esteem is often a sign of plasticity, indicating susceptibility to social dynamics, more exploratory and flexible cognitive styles.

### Error dynamics and adaptivity (metacognitive)

The ability to control cognitive states and behaviors aligns with sociocultural norms, and expectations are crucial for an individual’s adaptability. Precision in beliefs about oneself could influence the confidence one has in their self-model, guiding social interactions and emotional responses to social feedback. Within this context, the AIF postulates that valence reflects the goodness of an agent’s world model or the degree to which a self-prediction allows the system (e.g. a person or an animal) to be adaptive to its ecological niche. This self-prediction can be thought of as a prediction about what actions the system will take in a particular situation. Accurate self-predictions indicate that the system is capable of taking appropriate actions that align with its goals. Conversely, inaccurate self-predictions suggest a misalignment with the current context, hindering goal achievement.

Valence plays a special role in this framework by tracking and leveraging changes in the self-estimated adaptivity or value of the system. Valence refers to the positive or negative emotional value associated with a particular outcome or experience. In the context of active inference, valence can be thought of as a measure of the pleasure or displeasure associated with the accuracy of self-predictions (Hesp et al., 2021). If a self-prediction is accurate, it may be associated with a positive valence, whereas if a self-prediction is not accurate, it may be associated with a negative valence.

According to Hesp (2021), valence is intricately linked to the concept of affective charge (AC), which provides a quantitative measure of the congruence between predicted and realized outcomes. Affective charge is defined as


(5) $$ \mathrm{AC}=\boldsymbol{\beta}-\bar{\boldsymbol{\beta}}=(\pi-\bar{\pi})\cdot\boldsymbol{G}_{\pi}, $$

where *π* and $\bar{\pi}$ are inferred policies and the expected policies, respectively. The posterior belief over precision is denoted by $\bar{\boldsymbol{\gamma}}=1/\bar{\boldsymbol{\beta}}$. Affective charge acts as a reward prediction error signal within the system, encapsulating the system’s adaptability or its “goodness” of fit to environment. A positive affective charge indicates a low expected free energy, signifying successful subjective fitness and enhancing the agent’s confidence in its self-model. On the contrary, a negative affective charge, suggesting a high expected free energy, reflects on the prediction failure that may reduce the model’s perceived reliability. Consequently, individuals who are highly attuned to pleasure and displeasure in their emotional experiences tend to experience more significant fluctuations in self-esteem in response to social cues, echoing the active inference perspective where valence, as an index of the emotional response, reflects the adaptive accuracy of one’s self-model and predictions in social contexts (Pietromonaco and Barrett, 2009).

### Self and the niche

Agents use their generative model and gather evidence to predict the most optimal way to survive, but constantly starting from scratch would be energy-intensive (Friston, 2010). Therefore, they tend to gravitate toward environments that are most likely to support their survival (Albarracin et al., 2021). Agents in a social environment face tension as they try to acquire resources, which are not equally available to all members of the group (Constant et al., 2022). Some agents have more resources and can redistribute them, so being part of a group and having influence over its members increase the chances of accessing necessary resources for survival. This concept is often referred to as “group solidarity” or “social support.” For example, in his book “*The Structure of Social Action*,” sociologist Talcott Parsons (Parsons et al., 1949) writes that one of the primary functions of social systems is to provide for the material and non-material needs of their members. Similarly, in ‘*‘The Theory of Social and Economic Organization*,” sociologist Weber (2009) discusses the importance of social networks and relationships in facilitating access to resources. To be accepted into the group, agents must learn and follow the group’s norms (Constant et al., 2019). The generative model of the group determines which agents are favored (Jost and Banaji, 1994).

We thus posit that the self is constructed in relation to the niche. The self, as an emergent property of the brain’s interaction with its niche, is a construct that arises from this continuous dynamic. It is not a static entity but a process that is always in flux, adapting to the changing demands of the environment. The self is both shaped by and shapes its niche.

As a result, similar agents can be found in common places that have been marked by their presence (Vasil et al., 2020). Individual agents may have a limited effect on the environment, but a group of similar agents can collectively change the environment and turn it into an adaptive niche. These changes in the environment shape the possibilities available to the agent and provide outsourced information that the agent can use to synchronize with the group. As agents become more complex, the environment includes cultural niches, which are the combination of agents, conceptual fields, and their practices (Constant et al., 2019; Albarracin et al., 2022). An individual agent’s ability to read the cues of a cultural niche and navigate its practices determines their success in interacting with the niche and outsourcing processes to it (Constant et al., 2022; Albarracin et al., 2021). This back and forth between the agent and the environment leads to a synchronized state between their generative models (Albarracin et al., 2022). The changes in the agent and the environment continue over time, but they become synchronized in a non-equilibrium equilibrium state.

## Socially predicted self

Self-esteem works as an affective and cognitive evaluative tool regarding an individual’s overall worth and proficiency, as measured in the niche. Self-esteem can also provide, in normal circumstances, a reliable estimate of one’s fitness in his ecological niche, a fitness that can be appraised in terms of a gradient ranging from complete social inclusion to complete social exclusion. To cite the classic works of Leary et al. (1995): “a person’s feelings of self-esteem are an internal, subjective index or marker of the degree to which the individual is being included versus excluded by other people.” Thus, self-esteem, as they note, acts as a sociometer by providing an indicator of the perceived social fitness of the agent. Self-schema refers to the mental framework or set of expectations that individuals have about themselves, which can be influenced by social interactions and the expectations of others (Smith and Lewis, 1985).

Under active inference, the self is not only based on an individual’s own internal models and prediction errors but also influenced by social interactions and the expectations of others (Deane, 2021). Social interactions can adjust an individual’s self predictions through the process of precision weighting, which determines the relative importance of different sources of information in generating predictions (Albarracin et al., 2022). Precision weighting can be set socially, as individuals may place greater weight on the opinions and expectations of others compared to their own internal models and prediction errors.

The “ideal social self” refers to the idealized version of oneself that an individual strives to present to others in social situations (Kim and Hyun, 2013). This model can impact self-predictions by influencing an individual’s behavior and the way they perceive themselves in relation to others. For example, if an individual’s ideal social self is characterized by qualities such as confidence and assertiveness, they may behave in ways that are consistent with this model and adjust their self predictions accordingly.

The sociometer hypothesis has received much support throughout the years. For instance, Leary et al. (1998) demonstrated that self-esteem is negatively affected by real or fictitious cues of rejection. Individuals are tuned in to their social reality, as they are compared to others (Weisbuch et al., 2009). As we will see, this comparison is fundamentally important to the self and to behavior. The sociometer is a concept that refers to the internal monitoring system that individuals use to evaluate their social standing and the acceptance or rejection of their social group. This system can influence self-predictions by affecting an individual’s behavior and the way they perceive themselves in relation to others.

The self-image is generally internalized as the most accurate representation of the self. This self-image comes in part from our self-perception (Schröder-Abé et al., 2007a). We acquire elements of our self-perception from different sources. Interacting with others allows us to compare with our group and helps shape our self-concept (Salley et al., 2010). Individuals must model themselves in order to understand not only what they are going through but also what might be causing what they are going through and what actions influence the following outcomes. In psychology, this self-image, or self-schema, has been used to explain the way in which an individual integrates the social feedback they get (Ahadzadeh et al., 2017; Lewicki, 1984; Woolfolk et al., 1995). Self-schema essentially shows how information is integrated about the self in relation to its social desirability (understand social salience). Developing an accurate self-perception relies on how well we are capable of filtering and integrating information mirrored back to us by the external world.

In order to integrate information, an individual must be able to process feedback from their peers (Schröder-Abé et al., 2007b). When the group provides a stable environment for comparison, the individual may find it easier to create an accurate self-perception. Similarly, a homogeneous social group provides a potentially stable source of comparison, as many sources of comparison are more likely to be similar. Homogeneity of group members also offers the advantage that other group members are likely to be similar to the individual themselves. In this way, the individual may create a more accurate model of themselves, and the acceptable behaviors related to that model, as they will be more likely to observe these behaviors (Salley et al., 2010). By accurately modeling in their niche, the individual can better model themselves and arguably their optimal states.

### The background of norms

Internalizing norms allows us to present ourselves in a way that allows us to acquire resources from others (Pyszczynski et al., 2004). Power structures regulate and highlight these norms, which are the result of complex social structures with bias toward certain members of the group. Based on these norms, groups either include or reject members to various degrees (Salley et al., 2010). An individual’s self-model includes their understanding of their inclusion in the group, which is their self-esteem (Watson, 2017). However, the capacity to accurately compare oneself to the group may be impaired by factors such as mental health issues (Merianos et al., 2013), leading to maladaptive behaviors that reduce the individual’s ability to collaborate with the group and reinforce their exclusion from the necessary resources (Leary and Tangney, 2003; Leary et al., 1998; Witvliet et al., 2010).

Groups base their rejection on social ideals that have both internal and external reality (Constant et al., 2019). These ideals are stored in the generative models of individuals in the group and in the group itself. They can be manifested visually and promote specific goals that are useful for larger societal goals. These ideals are transmitted through mass media or network information and enforced by the social group. They are internalized, become second nature, and are used to define hierarchies in the group, with those farther from the ideal standing lower in the hierarchy (Bessenoff, 2006a).

Individuals develop a contextual, relative social persona based on a hierarchical comparison to the social ideal, which gives rise to their sense of self-worth (Ditzfeld and Showers, 2013). Comparing oneself to those who compare less favorably to the ideal leads to positive affect, while comparing oneself to those who compare more favorably leads to negative affect and a tendency to change oneself toward the ideal (Strahan et al., 2006; Zamani Sani et al., 2016; Reilly et al., 2014). For example, the ideal of thinness is often promoted as positive and desirable, leading to internalization of this ideal and the emergence of fatphobia and beauty ideals (Sender and Sullivan, 2008). Comparison to the ideal produces various degrees of error in an individual’s model of themselves and serves as a preference that helps improve their assessment of their relative position and value in the group (Tremblay et al., 2021; Constant et al., 2019).

An individual reduces variational free energy when they synchronize with their niche, reading the cues of other individuals in their social group to inform their actions and co-create a shared understanding of the world. This shared understanding, or generative model, spans across all members of the group and helps the individual to better predict and navigate their environment, improving their chances of survival. However, constantly updating their understanding of the world requires a lot of effort and energy. Motivation to change their self-concept must therefore be based on self-observation. This process can be understood as a hierarchical nested system, in which the individual not only models themselves but also assesses the precision of their model and chooses the appropriate course of action or updates their model of the world (Constant et al., 2019). Self-esteem, which is how we value ourselves in relation to societal expectations, can be thought of as a judgment about whether our actions are appropriate. This judgment takes into account our understanding of ourselves and how well we align with the ideals set by our social group. In other words, self-esteem is a measure of the accuracy of the individual’s understanding of their place in the group.

### Distance between the self and the social ideal

Comparing oneself to a social ideal has emotional and cognitive effects. Comparing poorly to the social ideal means that you are far from the prototype of the social category. We expect, from this distance, a certain type of social response. Beyond comparing to others, perceiving the self as discrepant with the ideal entails a set of negative affects. These negative affects can lead to a dissatisfaction with the self, along with chronic depression and the possibility of a social phobia (Bessenoff, 2006b).

The relationship between our self-perception and how far we are from societal ideals becomes more noticeable when we believe that we have control over how well we conform to these norms. They thus tend to perceive straying from the norm as a personal shortcoming, which can have an effect on their self-worth and self-esteem (Klaczynski et al., 2004). The emotional response to seeing oneself as lacking stems again from the expected social response of the group. Individuals tend to internalize expected reactions to straying and ideology around the locus of control for straying. Being removed from the social prototypes leads an individual to expect a social reaction of rejection based on the distance from the ideal (Bessenoff, 2006b). In contrast, people who are close to the social ideal tend to feel less compelled to change. In fact, people who do not feel like they are far from the social ideal do not feel that the signals relating to social comparison are as salient (Campbell, 1990). People with larger discrepancies feel a stronger salience of social cues related to comparison (Strahan et al., 2006). The salience of these cues justifies the emotional weight associated with the expected social response from the group. An individual is incentivized to conform to the group as they notice that they are straying from the norm.

### Social response to difference

Non-conformity to group norms can have serious social consequences, including rejection from the group and loss of access to resources. This is because groups tend to protect their integrity by punishing non-conformity, which is often perceived as uncooperative behavior (Holder, 1958; Willis, 1965). In order to maintain a higher likelihood of accessing resources, it is critical for individuals to have an accurate understanding of what the group values and how well they themselves fulfill these expectations. Even if an individual’s status in the group is relatively low, losing access to the group is a risk of losing access to all resources. As a result, individuals may find themselves caught in a trade-off between the awareness that they may receive little and the risk of receiving even less. Being able to predict one’s access to resources based on conformity and status in the group is important and can lead to more complex planning behaviors.

Self-esteem is a valuable tool in social regulation of the self, as it allows individuals to understand how well they might achieve a social outcome, either directly or through specific social reactions. The better matched an individual’s model of self is to the group’s expectations, the more likely they are to know whether their actions will lead to the desired outcome. In the final section, we will explore the role of valence as a second-order form of information in the predictive system that tracks and leverages changes in adaptivity. Valence, or the positivity or negativity of our self-model, is closely tied to self-esteem and can have significant effects on an individual’s well-being and overall functioning.

If an individual has an inaccurate model of the distance between their self-concept and the group’s expectations, they may act in a way that is rejected by the group. This rejection must be addressed and can signal to the individual that their model of the group may be erroneous, leading to a shift in self-esteem. Self-esteem can motivate the individual to change their behavior in response to this shift or to choose a course of action based on the expected response of the group. Negative emotions like anxiety can also influence behavior, causing individuals to avoid actions that may lead to rejection or social disapproval. In this way, self-esteem and negative emotions can guide individuals toward behaviors that are acceptable or desirable within their social group (Pyszczynski et al., 2004).

### Emotion as an indication of fitness

Changes in the degree to which self-predictions can be realized or achieved are felt as emotions, and these emotions drive behaviors in ways meant to improve the system’s predictive grip or accuracy (Hesp et al., 2021). Essentially, the system is motivated to take actions that will lead to more accurate self-predictions and a higher level of adaptivity. These emotions or feelings provide the system with a constant stream of second-order information about its own adaptivity in a particular niche or environment. This information can be used to guide the system’s actions and improve its ability to navigate and adapt to its environment.

Self-esteem can be understood in terms of predictive processing, a framework which posits that the brain builds generative models of the causes of its interoceptive and exteroceptive inputs in order to make predictions about future states of the body and the environment Barrett (2017). According to Barrett (2017), emotions are the felt (pre-reflectively conscious) activity of these generative models as they work to achieve allostasis or the regulation of the body’s homeostasis (the maintenance of vital variables within viable ranges). Allostasis can involve both reactive and predictive processes; for example, a high-level generative model may predict that night is about to fall and trigger a predictive allostasis that increases activity to keep the body warm. Emotional valence, in this context, refers to whether the generative models are successful in minimizing free energy (positive valence) or not (negative valence) (Joffily and Coricelli, 2013). By taking the first derivative of an organism’s free energy function (i.e. how free energy, as measured by prediction error, changes as a function of the activity of the organism’s generative models), one can determine the speed and direction of its change. Following Damasio, valence can be understood as the pre-reflectively felt outcome of these changes, with increases being felt as negative affects and minimizations as positive affects.

Anxiety is a negative emotion that can be understood as an increase in free energy (negative affect), resulting from activations of generative models predicting future increases in free energy. Pyszczynski et al. (2004) suggest that self-esteem can “buffer against” these predictions by altering the content of the self-model (Tremblay et al., 2021) and reducing the precision of interoceptive predictions. Too much self-esteem can be harmful because it may lead to a lack of negative predictions, while too little self-esteem can cause constant anxiety due to excessive negative predictions. In the final section, the AIF will be used to model the dynamics of self-esteem, examining the complex interactions between the self and the social world.

## Self-esteem as predicted adaptivity

Self-esteem can be thought of as a prediction about the self-model’s capacity to facilitate adaptation to the socio-cultural niche or environment in which an individual is embedded. In the framework of active inference, the self-model is a predictive model of the individual’s actions and experiences. This model adheres to the constraints imposed by the variational free energy and expected free energy concepts. The variational free energy, as represented in Equation 1, quantifies the efficacy of the biological agent’s internal model in relating latent states to sensory observations and deducing the dynamics of the environment. If the self-model is accurate and able to make good predictions, it means that the individual is able to take actions that are well-suited to their environment and that allow them to achieve their goals. On the other hand, if the self-model is not accurate, it means that the individual is not able to take actions that are well-suited to their environment and that hinder their ability to achieve their goals.

One way to tell if the self-model is succeeding is to use social feedback to estimate uncertainty about the self-model’s predictions. This pertains to the expected free energy (Eq. 2), which determines a prospective future course of actions by weighing the optimization of external values and the knowledge value. This feedback seeks to achieve a balance between maximizing extrinsic value and minimizing uncertainty through epistemic value. Social feedback can come in the form of responses from others to the individual’s actions or behaviors. If others respond positively to an individual’s actions, it can be taken as an indication that the self-model’s predictions are accurate and that the individual is succeeding in their environment. On the other hand, if others respond negatively to an individual’s actions, it can be taken as an indication that the self-model’s predictions are not accurate and that the individual is not succeeding in their environment.

In this framework, the agent applies a mean field approximation to form beliefs about hidden states. These beliefs are formed based on proprioceptive and exteroceptive information.

Socially related errors or mistakes can be extremely difficult to reduce precision on or correct. This is because such errors can have significant consequences for an individual’s social value or standing within their community. In our ancestral past, loss of social value could potentially have been a death sentence, as it would have meant being ostracized from the group and being unable to access the resources and protection that the group provided. As a result, changes in how well an individual is doing relative to predictions that are essential to their socially embedded self-model can have a huge impact on their self-organization or overall sense of self.

Mood disorders like depression can be seen as learned beliefs about a person’s inability to effectively reduce errors or manage them adaptively. In other words, depression may be seen as a learned belief that the individual is not able to effectively navigate their environment and achieve their goals, despite evidence to the contrary. This learned prior can then lead to changes in valence or the emotional value associated with outcomes, as the individual becomes less confident in their ability to be adaptive.

Self-esteem can be viewed as a prediction of an individual’s ability to adapt to their social and cultural environment. This prediction is shaped by social feedback and the individual’s personal experiences of success or failure in achieving their goals, as encoded in the expected free energy.

### A generative model of self-esteem

In this section, we present a hierarchical generative model of a self-organizing individual, equipped with advanced beliefs about themselves and their self-esteem. The key aspect of the dynamics of self-esteem is in the minimization of variational free energy and balancing of expected free energy. In line with the Self-Organizing Self-Esteem model (De Ruiter et al., 2017b), we propose a nested temporal structure where, at the lower level, an agent experiences moment-to-moment variation of self-esteem states based on sensory observation, and at a higher hierarchical level, more stable trait self-esteem is present. The agent minimizes the variational free energy bound on surprise by inferring the beliefs about ideal image state of self in his/her niche as well as self-perception of their overall qualities which are consistent with her sensory observations and his/her prior preferences, and the agent adopts policies that maximize the likelihood of attaining his/her preferred outcomes. We include core affective state of valence, in particular, in the high level of our hierarchical model since high/low self-esteem is directly related to higher well-being or anxiety/depression.

The hidden state of ideal image has three components: the first one is a sense of personal efficacy, namely, competence; the second component is social conformity that can be rooted in an adaptive strategy to increase fitness by imitating the behavior or following deontic cues in social niche; and finally, the last one is physical attractiveness which prompts reproductive fitness and promoting social identity. Note that physical attractiveness is only one of the possible signals an agent may be attuned to. In our model, we used this possibility because there is extensive literature documenting the importance of physical image in self esteem (Jung, 2006; KavehFarsani et al., 2020; Kostanski and Gullone, 1998; Olivardia et al., 2004), but we could have referred to many other factors which are self-reflexive.

Another set of latent states which can be inferred from observation of the environment is self-image. The self-concept or self-image is the set of beliefs that one holds about oneself as he/she interacts with the world. The self-esteem is considered as evaluating the self-concept in terms of identities and attributes that one develops through the process of role-taking in social constructs, and it can be integrated in terms of different components.

Another important factor is the sense of agency, which can be understood as an individual’s motivation and belief in their ability to intentionally act and pursue specific goals in the world. In contrast, a negative self-concept can lead to feelings of powerlessness and self-alienation, which occur when individuals feel they have lost control over the direction of their lives. The self-alienation can be felt when one perceives a sense of inauthenticity, while he/she engages in a decision-making process (Seto and Hicks, 2016). Uncertainty about self-concept stems in the lower congruent between perception of oneself and ideal self that will cause discontinuity of motivational patterns of behavior, which means the need to change the identity constantly and evinces anxiety or avoiding social disapproval situations. Low self-concept in an individual might decrease the accuracy of predicting sequence of actions, which was involved in social interaction. The anxiety originates because one avoids jeopardizing the concept of self by taking new signals from the environment and assimilating them, which might cause conflict with self-theory. In more severe cases, it could result in learned helplessness, a key symptom of depression wherein agents believe that they do not have control over events in their life in general and therefore interact less with their environment (Castiello et al., 2020).

## Further considerations and conclusion

### Potential computational experiments

The AIF for self-esteem that we just presented offers a powerful tool for simulating complex psychological phenomena, including the dynamics of self-esteem. Here, we outline several potential applications that could further our understanding of self-esteem’s role in various social and psychological contexts.

The first promising avenue for future research involves using our self-esteem model to simulate the influence of self-esteem on social interactions. This could entail developing a model in which agents with different levels of self-esteem interact, allowing us to observe how self-esteem influences behavior and interaction outcomes. Such a model could provide valuable insights into the social dynamics of self-esteem, including how it shapes interpersonal relationships and social networks.

This model could also be used to simulate the developmental trajectory of self-esteem in response to environmental factors. By creating a model in which agents progress through different life stages, we could observe how self-esteem develops in response to successes and failures. This could shed light on the critical factors that contribute to the development of self-esteem during childhood and adolescence and how these early experiences shape self-esteem in adulthood.

Another potential application involves simulating the influence of self-esteem on decision-making processes. By creating a model in which agents with different levels of self-esteem face various decision-making scenarios, we could observe how self-esteem influences choices and decision outcomes. This could provide a deeper understanding of the cognitive mechanisms through which self-esteem influences decision-making and how these processes contribute to the real-world consequences of self-esteem.

This active inference model could also be used to simulate the role of self-esteem in the development of mood disorders such as depression. By creating a model in which agents with varying levels of self-esteem are exposed to different stressors, we could observe how self-esteem influences emotional responses and the risk of developing mood disorders. This could provide valuable insights into the psychological mechanisms linking self-esteem and mental health and could inform interventions aimed at improving self-esteem to prevent or treat mood disorders.

Finally, our model could be used to simulate the influence of self-esteem on an individual’s ability to adapt to their environment. By creating a model in which agents with different levels of self-esteem are placed in different environments, we could observe how self-esteem influences adaptability. This could shed light on the role of self-esteem in resilience and coping and how it contributes to individuals’ ability to navigate and thrive in their environments.

### Misfiring of self-esteem

In the section on potential computational experiments, we laid out how self-esteem acts as a model of the self valence in a social niche. This model could allow us to explain in detail the various processes underlying self-esteem fluctuations and development in agents with adaptive strategies. By embedding ourselves in our group, we benefit from all the information and resources amassed by the niche. But our compliance to the group norm ensures its survival, and we are likely to be removed from the group if we stray too far from its norms. Having internalized this model of the group, individuals can appraise their likelihood of maintaining access to the resources and certainty around the group’s processes. Observing the self via self-esteem entails that the self-esteem encompasses signals from the self and interprets the data into a higher valence model. But it also acts as a prior. Self-esteem will allow the individual to predict the lower level states. The individual will infer the accuracy of their self-concept in relation to the social expectations and will infer the lower levels based on this assessment. However, self-esteem is subject to biases. By this, we mean that self-esteem can sometimes model reality inaccurately or take in some signals with a poor accuracy rating in relation to improving the agent’s fitness in the group. These biases can either keep self-esteem high or keep it low. If we focus on high self-esteem, there are many examples of perception shifting the way we integrate new information about ourselves into our self-esteem.

Self-esteem is positively related to everything from academic success (Li et al., 2018), life satisfaction (Perez-Fuentes et al., 2019), relationship satisfaction(Yildiz and Baytemir, 2016), and positive body image (KavehFarsani et al., 2020) to general well-being (Callea et al., 2019). Alternatively, low self-esteem is negatively associated with depression (Olivardia et al., 2004) , anxiety (Kostanski and Gullone, 1998; Olivardia et al., 2004), suicidal ideation (Sharaf et al., 2009), exhaustion (Donnellan et al., 2005; Li et al., 2018), and various social (Hall-Lande et al., 2007) or psychological problems (Donnellan et al., 2005). Thus, self-esteem is a very important factor in psychological and behavioral functioning. Self-esteem, which is the evaluation of one’s worth or abilities, can be influenced by external signals or be more stable, depending on how it is rooted. If self-esteem is based on intrinsic evaluation, such as self-traits, it is more likely to be stable over time. External signals, such as achievements or feedback, may have a greater impact on self-esteem if it is less stable. The source and relevance of the external signals also play a role in their impact on self-esteem. For example, if the signals come from someone who is considered emotionally relevant or comparable to the individual, they may be perceived as more accurate. An individual’s worldview can also affect how external signals are interpreted and integrated into self-esteem. If an individual holds a worldview that expects lower signals, they may not perceive them as accurate indicators of their place in society. On the other hand, if an individual believes in meritocracy, they may perceive discrimination as a signal that they are unfit in society and it will impact their self-esteem. (Major et al., 2007). These status-related beliefs can influence an individual’s ability to adapt to their social group and accurately assess their access to social and material value. When a model is resistant to change, it may be because the strong priors of the model act as top-down predictions and the individual is more likely to integrate signals that confirm their priors and less susceptible to signals that contradict them (Pyszczynski et al., 2004).

Self-esteem and self-concept are related, with self-esteem being at the top of the hierarchy and behaviors at the bottom in some models (Trautwein et al., 2006). Narcissistic personality disorders often involve a violent need to defend the self due to the fragility of the individual’s self-esteem, while a hypo-egoic mindset means that the individual is less in tune with their connection to the social group and more focused on concrete elements in the present moment, with less ability to make inferences about the future (Leary and Tangney, 2003). This state not only can help limit negative feedback and distress because the individual is unaware of being evaluated but also means that external events have little effect on the self-concept because abstract threats have little concrete implications for actions (Leary and Tangney, 2003; Leary and Allen, 2011). Our findings resonate with the Self-Organizing Self-Esteem model (De Ruiter et al., 2017b), which also describes similar processes of how self-esteem dynamics form nested hierarchies and how a subject and their environment become synchronized. Future research could further explore this alignment and the implications it has for understanding self-esteem.

### Limitations

A current limitation of this model is that self-esteem can be domain specific and is thus likely to change given the domain. The specific domain of competence may influence how important self-esteem is. The self-concept changes in relation to a specific context, which tends toward a social goal. If the self-concept corresponds more closely to the social ideal, the prior of self-esteem is given less precision as a prior. For instance, our model considers the importance of physical appearance, but this was simply an example. But not all domains value physical appearance at all, such as skill-based domains. The specific elements considered in self-image will be related to the kinds of tasks an individual has to enact and the kinds of metrics the individual is measured against. It is also currently difficult to assess how these different models become especially salient or how they interact to form a larger picture and overall sense of self-esteem. We can hypothesize, given our understanding of contextual effects on personality (Webster and Ward, 2011; Sinn and Moltschaniwskyj, 2005), that different priors for specific domain-specific models are instantiated given contextual cues. Meta-cognitive functions may help an individual assess which context is more likely to lead to the best fitness and direct individuals to seek out such niches where their fitness is maximized (Aunger and Curtis, 2013; Constant et al., 2022). Future work should test these hypotheses through simulation, attempting to replicate behaviors linked to high and low self-esteem. It should also investigate the complicated structures which lead to context-dependent self-esteem and the various ways in which some niches are more likely to produce high or low self-esteem.

To summarize, self-esteem plays a crucial role in the way that individuals perceive and interact with the world. It serves as a sociometer, allowing individuals to interpret and act on their standing in their social group. Through the use of active inference, we can better understand the computational mechanisms by which external factors impact self-esteem and how self-esteem can shape an individual’s behavior. Additionally, examining self-esteem through the lens of active inference allows us to understand the role that prior beliefs and confirmation biases play in shaping an individual’s self-esteem. Understanding self-esteem and its influence on behavior is important for improving mental health and well-being. Active inference provides a more comprehensive formalization of self-esteem as its domain is generalisable and describes the computational mechanisms, whereas previous models focused on domain-specific circumstances (such as social inclusion vs exclusion) and provided a conceptual account of how self-esteem develops but not highlighting the actual underlying mechanisms of the calculation.
